# 2-(2,4,6-Trimethyl­phen­yl)-1,10-phenanthroline

**DOI:** 10.1107/S1600536809010800

**Published:** 2009-04-01

**Authors:** Yuxin Zhao, Yongping Zhang, Peiju Yang, Biao Wu

**Affiliations:** a State Key Laboratory of Applied Organic Chemistry, College of Chemistry and Chemical Engineering, Lanzhou University, Lanzhou 730000, People’s Republic of China; b State Key Laboratory for Oxo Synthesis & Selective Oxidation, Lanzhou Institute of Chemical Physics, CAS, Lanzhou 730000, People’s Republic of China

## Abstract

In the title mol­ecule, C_21_H_18_N_2_, the mean plane of the benzene ring of the mesityl group forms a dihedral angle of 82.69 (4)° with that of the phenanthroline ring system. The crystal structure is stabilized by π–π stacking inter­actions between the phenanthroline system and the benzene ring of the mesityl group of a symmetry-related mol­ecule, with centroid–centroid distances of 3.7776 (14) and 3.7155 (13) Å.

## Related literature

For background information on phenanthroline derivatives, see: Schmittel *et al.* (2001 ▶); Garas & Vagg (2000 ▶). For information on phenanthroline ligands as used in coordination chemistry, see: Sauvage (1990 ▶). For the synthetic procedure, see: Schmittel & Ganz (1997 ▶).
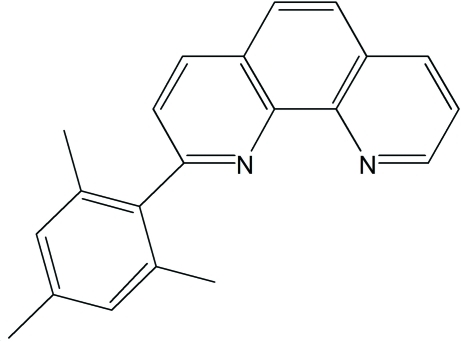


## Experimental

### 

#### Crystal data

C_21_H_18_N_2_
*M* *_r_* = 298.37
Monoclinic, 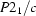

*a* = 14.5778 (19) Å
*b* = 8.9877 (11) Å
*c* = 13.5790 (11) Å
β = 112.166 (4)°
*V* = 1647.6 (3) Å^3^
*Z* = 4
Mo *K*α radiation
μ = 0.07 mm^−1^
*T* = 293 K
0.35 × 0.23 × 0.19 mm

#### Data collection

Bruker SMART area-detector diffractometer
Absorption correction: multi-scan (*SADABS*; Sheldrick, 1996 ▶) *T*_min_ = 0.976, *T*_max_ = 0.987
9474 measured reflections
3511 independent reflections
2150 reflections with *I* > 2σ(*I*)
*R*_int_ = 0.031

#### Refinement

*R*[*F* ^2^ > 2σ(*F* ^2^)] = 0.057
*wR*(*F* ^2^) = 0.181
*S* = 1.06
3511 reflections
208 parameters
H-atom parameters constrained
Δρ_max_ = 0.24 e Å^−3^
Δρ_min_ = −0.19 e Å^−3^

### 

Data collection: *SMART* (Bruker, 1998 ▶); cell refinement: *SAINT* (Bruker, 1998 ▶); data reduction: *SAINT*; program(s) used to solve structure: *SHELXS97* (Sheldrick, 2008 ▶); program(s) used to refine structure: *SHELXL97* (Sheldrick, 2008 ▶); molecular graphics: *ORTEP-3 for Windows* (Farrugia, 1999 ▶); software used to prepare material for publication: *SHELXL97*.

## Supplementary Material

Crystal structure: contains datablocks I, global. DOI: 10.1107/S1600536809010800/lh2789sup1.cif

Structure factors: contains datablocks I. DOI: 10.1107/S1600536809010800/lh2789Isup2.hkl

Additional supplementary materials:  crystallographic information; 3D view; checkCIF report

## Figures and Tables

**Fig. 1.**
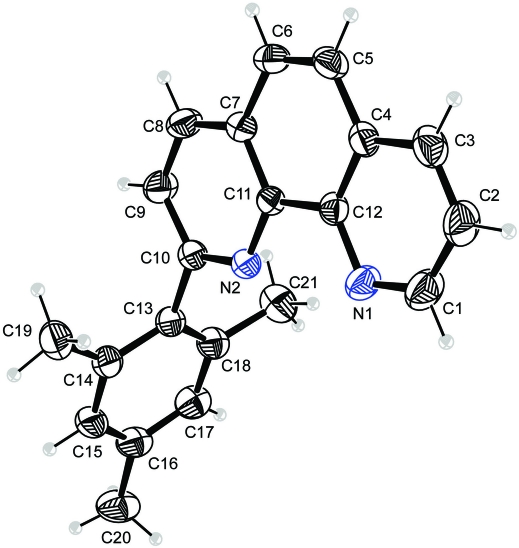
The molecular structure of the title compound, with the atom-numbering scheme. Displacement ellipsoids are drawn at the 30% probability level.

**Fig. 2.**
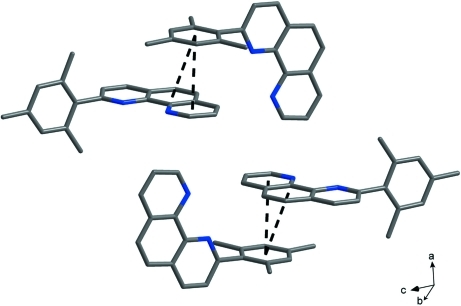
Part of the crystal structure showing the /p-/p stacking interactions (dashed lines) between the phenanthroline ring system and symmetry related benzene rings. H atoms have been omitted for clarity.

## supplementary crystallographic information

### Comment

Phenanthroline derivatives are well known nitrogen-containing heterocyclic compounds, and their syntheses have been extensively studied (Garas & Vagg 2000, Schmittel *et al.*, 2001). 1,10-Phenanthroline ligands have been widely used in transition metal coordination chemistry because their steric and electronic environment can be conveniently tailored by varying the substituents (Sauvage, 1990). We have therefore synthesized a series of 2-substituted-1,10-phenanthrolines including the title compound to study their applications in metal coordination chemistry.

The molecular structure of the title compound is shown in Fig. 1. The benzene ring of the  mesityl group forms a dihedral angle of 82.69 (4)° with the mean-plane of phenanthroline ring system. The crystal structure is stabilized by intermolecular π-π stacking interactions between the phenanthroline moiety and the benzene ring of a symmetry related molecule (Fig. 2) where Cg1···Cg2 and Cg2···Cg3 are 3.7776 (14)Å and 3.7155 (13)Å, respectively (with the perpendicaular distances for each being ca. 3.5Å). Cg1, Cg2 and Cg3 are the centroids defined by ring atoms N1/C1-C4/C12, C13-C18 and C4-C12, respectively.

### Experimental

The title compound was synthesized according to a reported literature procedures (Schmittel & Ganz 1997). Crystals suitable for X-ray diffraction were obtained  evaporation of a solution of the title compound in ethyl acetate.

### Refinement

All H atoms attached to C atoms were fixed geometrically and treated as riding with C—H = 0.96 Å (methyl), and 0.93 Å (aromatic) with *U*iso(H) = 1.2Ueq(C) or *U*_iso_(H) = 1.5U_eq_(methyl).

### Crystal data

**Table:**

C_21_H_18_N_2_	*F*(000) = 632
*M**_r_* = 298.37	*D*_x_ = 1.203 Mg m^−^^3^
Monoclinic, *P*2_1_/*c*	Mo *K*α radiation, λ = 0.71073 Å
Hall symbol: -P 2ybc	Cell parameters from 2140 reflections
*a* = 14.5778 (19) Å	θ = 2.7–25.4°
*b* = 8.9877 (11) Å	µ = 0.07 mm^−^^1^
*c* = 13.5790 (11) Å	*T* = 293 K
β = 112.166 (4)°	Block, colourless
*V* = 1647.6 (3) Å^3^	0.35 × 0.23 × 0.19 mm
*Z* = 4	

### Data collection

**Table:**

Bruker APEXII area-detector diffractometer	3511 independent reflections
Radiation source: fine-focus sealed tube	2150 reflections with *I* > 2σ(*I*)
graphite	*R*_int_ = 0.031
ω scans	θ_max_ = 26.9°, θ_min_ = 2.7°
Absorption correction: multi-scan (*SADABS*; Sheldrick, 1996)	*h* = −18→17
*T*_min_ = 0.976, *T*_max_ = 0.987	*k* = −11→10
9474 measured reflections	*l* = −17→15

### Refinement

**Table:**

Refinement on *F*^2^	Primary atom site location: structure-invariant direct methods
Least-squares matrix: full	Secondary atom site location: difference Fourier map
*R*[*F*^2^ > 2σ(*F*^2^)] = 0.057	Hydrogen site location: inferred from neighbouring sites
*wR*(*F*^2^) = 0.181	H-atom parameters constrained
*S* = 1.06	*w* = 1/[σ^2^(*F*_o_^2^) + (0.084*P*)^2^ + 0.1669*P*] where *P* = (*F*_o_^2^ + 2*F*_c_^2^)/3
3511 reflections	(Δ/σ)_max_ < 0.001
208 parameters	Δρ_max_ = 0.24 e Å^−^^3^
0 restraints	Δρ_min_ = −0.19 e Å^−^^3^

### Special details

**Table:**

Geometry. All e.s.d.'s (except the e.s.d. in the dihedral angle between two l.s. planes) are estimated using the full covariance matrix. The cell e.s.d.'s are taken into account individually in the estimation of e.s.d.'s in distances, angles and torsion angles; correlations between e.s.d.'s in cell parameters are only used when they are defined by crystal symmetry. An approximate (isotropic) treatment of cell e.s.d.'s is used for estimating e.s.d.'s involving l.s. planes.
Refinement. Refinement of *F*^2^ against ALL reflections. The weighted *R*-factor *wR* and goodness of fit *S* are based on *F*^2^, conventional *R*-factors *R* are based on *F*, with *F* set to zero for negative *F*^2^. The threshold expression of *F*^2^ > σ(*F*^2^) is used only for calculating *R*-factors(gt) *etc*. and is not relevant to the choice of reflections for refinement. *R*-factors based on *F*^2^ are statistically about twice as large as those based on *F*, and *R*- factors based on ALL data will be even larger.

### Fractional atomic coordinates and isotropic or equivalent isotropic displacement parameters (Å^2^)

**Table:**

	*x*	*y*	*z*	*U*_iso_*/*U*_eq_	
N1	0.09525 (12)	0.20234 (19)	0.35983 (13)	0.0639 (5)	
N2	0.23560 (10)	0.39234 (16)	0.49200 (11)	0.0503 (4)	
C1	0.02762 (16)	0.1138 (3)	0.29282 (19)	0.0802 (7)	
H1A	−0.0102	0.0559	0.3200	0.096*	
C2	0.00892 (17)	0.1011 (3)	0.18436 (19)	0.0830 (7)	
H2A	−0.0401	0.0373	0.1414	0.100*	
C3	0.06359 (15)	0.1835 (3)	0.14320 (17)	0.0709 (6)	
H3A	0.0527	0.1771	0.0713	0.085*	
C4	0.13668 (13)	0.2784 (2)	0.21015 (14)	0.0527 (5)	
C5	0.19644 (15)	0.3666 (2)	0.17047 (15)	0.0605 (5)	
H5A	0.1874	0.3607	0.0990	0.073*	
C6	0.26533 (15)	0.4579 (2)	0.23519 (16)	0.0617 (5)	
H6A	0.3030	0.5157	0.2077	0.074*	
C7	0.28227 (13)	0.4683 (2)	0.34577 (14)	0.0513 (5)	
C8	0.35443 (15)	0.5618 (2)	0.41600 (16)	0.0668 (6)	
H8A	0.3941	0.6200	0.3915	0.080*	
C9	0.36619 (15)	0.5671 (2)	0.52044 (16)	0.0672 (6)	
H9A	0.4153	0.6266	0.5679	0.081*	
C10	0.30418 (13)	0.4828 (2)	0.55576 (14)	0.0518 (5)	
C11	0.22481 (12)	0.38370 (19)	0.38836 (13)	0.0449 (4)	
C12	0.14971 (12)	0.2850 (2)	0.31808 (14)	0.0486 (5)	
C13	0.31208 (14)	0.4929 (2)	0.66863 (14)	0.0544 (5)	
C14	0.38347 (14)	0.4096 (2)	0.74841 (16)	0.0627 (6)	
C15	0.38632 (16)	0.4189 (3)	0.85174 (16)	0.0690 (6)	
H15A	0.4337	0.3638	0.9048	0.083*	
C16	0.32177 (18)	0.5063 (3)	0.87862 (16)	0.0703 (6)	
C17	0.25303 (17)	0.5879 (2)	0.79860 (17)	0.0704 (6)	
H17A	0.2094	0.6485	0.8156	0.085*	
C18	0.24611 (15)	0.5836 (2)	0.69393 (16)	0.0622 (5)	
C19	0.45479 (16)	0.3098 (3)	0.72337 (19)	0.0877 (8)	
H19B	0.4980	0.2623	0.7874	0.132*	
H19C	0.4933	0.3679	0.6937	0.132*	
H19D	0.4185	0.2354	0.6730	0.132*	
C20	0.16739 (18)	0.6713 (3)	0.6093 (2)	0.0847 (7)	
H20B	0.1293	0.7268	0.6409	0.127*	
H20C	0.1246	0.6045	0.5568	0.127*	
H20D	0.1980	0.7387	0.5761	0.127*	
C21	0.3232 (2)	0.5084 (3)	0.99054 (18)	0.0934 (8)	
H21B	0.3756	0.4453	1.0352	0.140*	
H21C	0.2609	0.4729	0.9900	0.140*	
H21D	0.3341	0.6083	1.0175	0.140*	

### Atomic displacement parameters (Å^2^)

**Table:**

	*U*^11^	*U*^22^	*U*^33^	*U*^12^	*U*^13^	*U*^23^
N1	0.0589 (9)	0.0730 (11)	0.0617 (10)	−0.0201 (8)	0.0249 (9)	−0.0090 (9)
N2	0.0487 (8)	0.0586 (9)	0.0419 (8)	−0.0056 (7)	0.0153 (7)	−0.0014 (7)
C1	0.0678 (13)	0.0949 (17)	0.0796 (16)	−0.0298 (13)	0.0299 (13)	−0.0175 (14)
C2	0.0660 (14)	0.1019 (19)	0.0699 (15)	−0.0276 (13)	0.0131 (12)	−0.0222 (14)
C3	0.0640 (13)	0.0850 (15)	0.0522 (12)	−0.0036 (12)	0.0088 (11)	−0.0116 (11)
C4	0.0469 (10)	0.0614 (11)	0.0446 (10)	0.0044 (8)	0.0112 (8)	−0.0032 (9)
C5	0.0663 (12)	0.0735 (13)	0.0413 (10)	0.0042 (10)	0.0197 (10)	0.0016 (10)
C6	0.0669 (12)	0.0732 (13)	0.0515 (12)	−0.0060 (11)	0.0299 (10)	0.0040 (10)
C7	0.0512 (10)	0.0565 (11)	0.0459 (10)	−0.0018 (8)	0.0181 (9)	0.0017 (9)
C8	0.0672 (13)	0.0771 (14)	0.0613 (13)	−0.0227 (11)	0.0303 (11)	−0.0009 (11)
C9	0.0650 (12)	0.0785 (14)	0.0557 (12)	−0.0278 (11)	0.0201 (10)	−0.0117 (11)
C10	0.0498 (10)	0.0587 (11)	0.0440 (10)	−0.0050 (9)	0.0146 (9)	−0.0012 (9)
C11	0.0427 (9)	0.0509 (10)	0.0409 (10)	0.0023 (7)	0.0156 (8)	0.0014 (8)
C12	0.0421 (9)	0.0537 (10)	0.0478 (11)	0.0038 (8)	0.0145 (8)	−0.0013 (8)
C13	0.0530 (10)	0.0630 (12)	0.0425 (10)	−0.0142 (9)	0.0127 (9)	−0.0060 (9)
C14	0.0535 (11)	0.0804 (14)	0.0493 (12)	−0.0108 (10)	0.0138 (10)	−0.0015 (10)
C15	0.0664 (13)	0.0866 (15)	0.0448 (12)	−0.0146 (11)	0.0105 (10)	0.0030 (11)
C16	0.0823 (15)	0.0817 (15)	0.0463 (12)	−0.0292 (13)	0.0234 (12)	−0.0135 (11)
C17	0.0813 (15)	0.0749 (14)	0.0602 (13)	−0.0117 (12)	0.0325 (12)	−0.0173 (11)
C18	0.0672 (13)	0.0644 (12)	0.0523 (12)	−0.0087 (10)	0.0194 (10)	−0.0075 (10)
C19	0.0653 (14)	0.128 (2)	0.0650 (15)	0.0148 (14)	0.0187 (12)	0.0125 (14)
C20	0.0911 (16)	0.0851 (16)	0.0751 (16)	0.0156 (14)	0.0281 (14)	−0.0010 (13)
C21	0.124 (2)	0.1078 (19)	0.0529 (13)	−0.0323 (17)	0.0386 (14)	−0.0142 (13)

### Geometric parameters (Å, °)

**Table:**

N1—C1	1.325 (3)	C10—C13	1.496 (3)
N1—C12	1.357 (2)	C11—C12	1.452 (2)
N2—C10	1.325 (2)	C13—C18	1.399 (3)
N2—C11	1.358 (2)	C13—C14	1.402 (3)
C1—C2	1.398 (3)	C14—C15	1.391 (3)
C1—H1A	0.9300	C14—C19	1.506 (3)
C2—C3	1.353 (3)	C15—C16	1.377 (3)
C2—H2A	0.9300	C15—H15A	0.9300
C3—C4	1.400 (3)	C16—C17	1.379 (3)
C3—H3A	0.9300	C16—C21	1.512 (3)
C4—C12	1.406 (2)	C17—C18	1.387 (3)
C4—C5	1.426 (3)	C17—H17A	0.9300
C5—C6	1.337 (3)	C18—C20	1.505 (3)
C5—H5A	0.9300	C19—H19B	0.9600
C6—C7	1.430 (3)	C19—H19C	0.9600
C6—H6A	0.9300	C19—H19D	0.9600
C7—C8	1.402 (3)	C20—H20B	0.9600
C7—C11	1.407 (2)	C20—H20C	0.9600
C8—C9	1.364 (3)	C20—H20D	0.9600
C8—H8A	0.9300	C21—H21B	0.9600
C9—C10	1.396 (3)	C21—H21C	0.9600
C9—H9A	0.9300	C21—H21D	0.9600
			
C1—N1—C12	116.26 (18)	C4—C12—C11	118.84 (16)
C10—N2—C11	118.42 (15)	C18—C13—C14	120.00 (18)
N1—C1—C2	124.9 (2)	C18—C13—C10	119.51 (17)
N1—C1—H1A	117.6	C14—C13—C10	120.47 (18)
C2—C1—H1A	117.6	C15—C14—C13	118.6 (2)
C3—C2—C1	118.6 (2)	C15—C14—C19	120.2 (2)
C3—C2—H2A	120.7	C13—C14—C19	121.19 (18)
C1—C2—H2A	120.7	C16—C15—C14	122.6 (2)
C2—C3—C4	119.2 (2)	C16—C15—H15A	118.7
C2—C3—H3A	120.4	C14—C15—H15A	118.7
C4—C3—H3A	120.4	C15—C16—C17	117.45 (19)
C3—C4—C12	118.22 (18)	C15—C16—C21	121.5 (2)
C3—C4—C5	121.17 (18)	C17—C16—C21	121.0 (2)
C12—C4—C5	120.61 (16)	C16—C17—C18	122.9 (2)
C6—C5—C4	120.49 (17)	C16—C17—H17A	118.6
C6—C5—H5A	119.8	C18—C17—H17A	118.6
C4—C5—H5A	119.8	C17—C18—C13	118.5 (2)
C5—C6—C7	121.31 (18)	C17—C18—C20	120.5 (2)
C5—C6—H6A	119.3	C13—C18—C20	120.96 (18)
C7—C6—H6A	119.3	C14—C19—H19B	109.5
C8—C7—C11	117.04 (17)	C14—C19—H19C	109.5
C8—C7—C6	122.79 (17)	H19B—C19—H19C	109.5
C11—C7—C6	120.17 (16)	C14—C19—H19D	109.5
C9—C8—C7	119.75 (18)	H19B—C19—H19D	109.5
C9—C8—H8A	120.1	H19C—C19—H19D	109.5
C7—C8—H8A	120.1	C18—C20—H20B	109.5
C8—C9—C10	119.64 (18)	C18—C20—H20C	109.5
C8—C9—H9A	120.2	H20B—C20—H20C	109.5
C10—C9—H9A	120.2	C18—C20—H20D	109.5
N2—C10—C9	122.36 (17)	H20B—C20—H20D	109.5
N2—C10—C13	117.01 (16)	H20C—C20—H20D	109.5
C9—C10—C13	120.63 (16)	C16—C21—H21B	109.5
N2—C11—C7	122.74 (16)	C16—C21—H21C	109.5
N2—C11—C12	118.68 (15)	H21B—C21—H21C	109.5
C7—C11—C12	118.57 (15)	C16—C21—H21D	109.5
N1—C12—C4	122.83 (16)	H21B—C21—H21D	109.5
N1—C12—C11	118.33 (16)	H21C—C21—H21D	109.5
			
C12—N1—C1—C2	−0.3 (3)	C3—C4—C12—C11	−179.59 (16)
N1—C1—C2—C3	0.5 (4)	C5—C4—C12—C11	0.3 (3)
C1—C2—C3—C4	0.0 (3)	N2—C11—C12—N1	−1.8 (2)
C2—C3—C4—C12	−0.5 (3)	C7—C11—C12—N1	179.26 (15)
C2—C3—C4—C5	179.6 (2)	N2—C11—C12—C4	178.47 (15)
C3—C4—C5—C6	179.35 (18)	C7—C11—C12—C4	−0.5 (2)
C12—C4—C5—C6	−0.5 (3)	N2—C10—C13—C18	−80.7 (2)
C4—C5—C6—C7	1.0 (3)	C9—C10—C13—C18	98.5 (2)
C5—C6—C7—C8	179.43 (19)	N2—C10—C13—C14	97.8 (2)
C5—C6—C7—C11	−1.2 (3)	C9—C10—C13—C14	−82.9 (2)
C11—C7—C8—C9	0.1 (3)	C18—C13—C14—C15	0.5 (3)
C6—C7—C8—C9	179.5 (2)	C10—C13—C14—C15	−178.08 (17)
C7—C8—C9—C10	−1.9 (3)	C18—C13—C14—C19	179.37 (18)
C11—N2—C10—C9	−1.0 (3)	C10—C13—C14—C19	0.8 (3)
C11—N2—C10—C13	178.28 (15)	C13—C14—C15—C16	0.1 (3)
C8—C9—C10—N2	2.5 (3)	C19—C14—C15—C16	−178.8 (2)
C8—C9—C10—C13	−176.75 (19)	C14—C15—C16—C17	−0.7 (3)
C10—N2—C11—C7	−1.0 (3)	C14—C15—C16—C21	176.9 (2)
C10—N2—C11—C12	−179.93 (15)	C15—C16—C17—C18	0.8 (3)
C8—C7—C11—N2	1.4 (3)	C21—C16—C17—C18	−176.9 (2)
C6—C7—C11—N2	−178.00 (16)	C16—C17—C18—C13	−0.2 (3)
C8—C7—C11—C12	−179.66 (16)	C16—C17—C18—C20	177.98 (19)
C6—C7—C11—C12	0.9 (3)	C14—C13—C18—C17	−0.4 (3)
C1—N1—C12—C4	−0.3 (3)	C10—C13—C18—C17	178.17 (17)
C1—N1—C12—C11	180.00 (17)	C14—C13—C18—C20	−178.62 (18)
C3—C4—C12—N1	0.7 (3)	C10—C13—C18—C20	0.0 (3)
C5—C4—C12—N1	−179.46 (16)		
